# Porcine Circovirus Type 2 Activates CaMMKβ to Initiate Autophagy in PK-15 Cells by Increasing Cytosolic Calcium

**DOI:** 10.3390/v8050135

**Published:** 2016-05-20

**Authors:** Yuanxing Gu, Baozhu Qi, Yingshan Zhou, Xiaowu Jiang, Xian Zhang, Xiaoliang Li, Weihuan Fang

**Affiliations:** Zhejiang Provincial Key Laboratory of Preventive Veterinary Medicine, Institute of Preventive Veterinary Medicine, Zhejiang University, Hangzhou 310058, China; guzi.123@163.com (Y.G.); qibaozhu@yeah.net (B.Q.); yszhou@zju.edu.cn (Y.Z.); cqrcxwj@163.com (X.J.); zhangxian073@163.com (X.Z.); xlli@zju.edu.cn (X.L.)

**Keywords:** porcine circovirus 2, autophagy, Ca^2+^ signaling, pathogenesis

## Abstract

Porcine circovirus type 2 (PCV2) induces autophagy via the 5′ adenosine monophosphate-activated protein kinase (AMPK)/extracellular signal-regulated kinase (ERK)/tuberous sclerosis complex 2 (TSC2)/mammalian target of rapamycin (mTOR) pathway in pig kidney PK-15 cells. However, the underlying mechanisms of AMPK activation in autophagy induction remain unknown. With specific inhibitors and RNA interference (RNAi), we show that PCV2 infection upregulated calcium/calmodulin-dependent protein kinase kinase-beta (CaMKKβ) by increasing cytosolic Ca^2+^ via inositol 1,4,5-trisphosphate receptor (IP3R). Elevation of cytosolic calcium ion (Ca^2+^) did not seem to involve inositol 1,4,5-trisphosphate (IP3) release from phosphatidylinositol 4,5-bisphosphate (PIP2) by phosphoinositide phospholipase C-gamma (PLC-γ). CaMKKβ then activated both AMPK and calcium/calmodulin-dependent protein kinase I (CaMKI). PCV2 employed CaMKI and Trp-Asp (WD) repeat domain phosphoinositide-interacting protein 1 (WIPI1) as another pathway additional to AMPK signaling in autophagy initiation. Our findings could help better understanding of the signaling pathways of autophagy induction as part of PCV2 pathogenesis. Further research is warranted to study if PCV2 interacts directly with IP3R or indirectly with the molecules that antagonize IP3R activity responsible for increased cytosolic Ca^2+^ both in PK-15 cells and PCV2-targeted primary cells from pigs.

## 1. Introduction

Porcine circovirus type 2 (PCV2) has emerged as one of the most important pathogens in pigs since its initial recognition in 1998 [1]. The clinical signs caused by PCV2 infection was first described as post-weaning multisystemic wasting syndrome (PMWS), and were collectively renamed porcine circovirus-associated disease (PCVAD) in the United States or porcine circovirus disease (PCVD) in Europe, since many more clinical diseases such as reproductive disorders, enteric diseases, and respiratory signs were linked to PCV2 infection [2]. To our knowledge, no specific antiviral therapy is available for PCV2 due to the poor understanding of pathogenesis.

PCV2 has a circular single-stranded DNA genome of about 1.7 kb that contains two major open reading frames (ORFs), ORF1 encoding the replicase proteins Rep and Rep’ essential for transcription, and ORF2 encoding the capsid (Cap) protein for genome packing [3,4]. Cap is an important immunogenic protein and contributes to autophagy induction and induces cell death in pig kidney PK-15 cells [5,6,7,8]. PCV2 might also express functional proteins of ORF3, ORF4, and ORF5. It has been reported that the ORF3 protein induced apoptosis by activating caspase-8 and caspase-3 [9], although it remains a controversial issue [2]. Proteins encoded by ORF4 and ORF5, although not essential for virus replication, appeared to be functional as well. ORF4 was found to be anti-apoptotic by suppressing caspases activity [10]. ORF5 was capable of inducing endoplasmic reticulum (ER) stress and nuclear factor kappa-light-chain-enhancer of activated B cells (NF-κB) activation [11]. Although encouraging progress has been made, the pathogenic mechanisms underlying PCV2 infection remain largely unknown. Recent studies indicate that PCV2 infection leads to thymocyte selection dysregulation and that its latency in the fetal thymi probably causes immune tolerance [12,13]. This suggests not only a challenge of effective vaccination against such latent pathogen, but also a need to better understand the mechanisms of PCV2 latency in the fetal thymus. One question is: does the virus hijack the autophagic machinery in favor of its latent survival?

Autophagy is a precisely regulated membrane-dependent process that secures cellular homeostasis and involves more than 30 proteins [14,15]. Viral infections could induce autophagic responses [16]. Such responses could be antiviral or proviral, depending on the types of viruses or of host cells and the cellular environments [17,18]. Calcium ion (Ca^2+^) is a ubiquitous intracellular messenger responsible for many important signal-transducing cellular processes such as gene transcription, differentiation, proliferation, and activation of kinases [19,20]. Ca^2+^ and Ca^2+^-sensor proteins likely play a dual role in autophagy regulation, depending on types of cells, cellular situation and Ca^2+^ signal [21,22]. Excessive cytosolic Ca^2+^ released from the ER has been reported to induce autophagy through calcium/calmodulin-dependent protein kinase kinase-beta (CaMKKβ)-mediated activation of 5′ adenosine monophosphate-activated protein kinase (AMPK) with subsequent inhibition of mammalian target of rapamycin (mTOR) [23,24]. CaMKKβ also activates calcium/calmodulin-dependent protein kinase I (CaMKI) which is involved in stimulation of the essential phosphatidylinositol 3-phosphate (PI3P) effector Trp-Asp (WD) repeat domain phosphoinositide-interacting protein 1 (WIPI1) [25,26,27]. WIPI1 can facilitate nucleation of isolated membrane structures from the ER at the early stage of the autophagic process [28]. Thus, the abundance of WIPI1 mRNA and the number of WIPI1-positive puncta have been proposed as an additional characteristics of autophagy [27,29,30].

Some viruses can manipulate the host Ca^2+^ signal to benefit their life cycles [31,32]. Hepatitis B virus (HBV) X protein targets the human B-cell lymphoma 2 (Bcl-2) homolog, apoptosis regulator CED-9, to induce cytosolic Ca^2+^ increase and cell death in *Caenorhabditis elegans* [33]. Ca^2+^ influx is necessary at the early infection events of West Nile virus (WNV) for its efficient replication [34]. Rotavirus-encoded viroporin nonstructural protein 4 (NSP4) releases Ca^2+^ from the ER into the cytosol, thus activating CaMKKβ signaling to initiate autophagy [35].

PCV2 could induce ER Ca^2+^ release through the inositol 1,4,5-trisphosphate receptor (IP3R) in lymphocytes of piglets, which was considered responsible for the apoptosis induction [36]. We have found that PCV2 could induce autophagy to facilitate its replication via the AMPK/ extracellular signal-regulated kinase (ERK)/ tuberous sclerosis complex 2 (TSC2)/mTOR pathway in PK-15 cells, but the mechanisms of AMPK activation remain unknown [7,37]. In the present study, we further investigated the molecules upstream of AMPK that are involved in autophagy induction in PK-15 cells infected with PCV2. Our results demonstrate that CaMKKβ is the upstream regulator of AMPK during PCV2 infection. CaMKKβ/CaMKI/WIPI1 is another pathway additional to AMPK signaling that contributes to PCV2-induced autophagy. Activation of CaMKKβ results from the increased cytosolic Ca^2+^ through IP3R.

## 2. Materials and Methods

### 2.1. Virus, Cell Lines, and Plasmids

A PCV2 isolate SY4 strain (PCV2b, GenBank accession no. GU325754) was originally isolated from the lung of a pig with naturally-occurring PCVAD in Zhejiang, China. We could not detect any porcine circovirus type 1 (PCV1) nor PCV2 contamination in our PK-15 cell lines. PK-15 cells stably expressing enhanced green fluorescent protein (EGFP)-tagged microtubule-associated protein 1 light chain 3 (LC3), referred to as PK-15/EGFP-LC3 cells, were maintained in our laboratory [7]. PK-15 cells were cultured at 37 °C and 5% CO_2_ in minimal essential medium (MEM) (HyClone, South Logan, UT, USA) supplemented with 10% newborn calf serum (Gibco, Grand Island, NY, USA), 1% l-glutamine, 1% non-essential amino acids (100×, GIBCO-BRL), 100 U/mL penicillin, and 100 μg/mL streptomycin. The pcDNA3.1 and pcDNA3.1-*Discosoma sp*. red fluorescent protein (DsRed) vectors (Invitrogen, Eugene, OR, USA) were used for construction of all eukaryotic expression vectors. The pcDNA3-TN-XXL vector (Addgene, Cambridge, MA, USA) containing a genetically encoded troponin C-based Ca^2+^ indicator TN-XXL that is fused to the enhanced cyan fluorescent protein (ECFP) and yellow fluorescent protein (YFP) in its N-terminal and C-terminal, respectively, can be used to examine cytosolic Ca2+ levels by fluorescence resonance energy transfer (FRET) [38].

### 2.2. Recombinant Plasmids, Small Interfering RNAs and Transfection

To construct DsRed-WIPI1 fusion vector (pcDNA3.1-DsRed-WIPI1), the porcine *WIPI1* gene (GenBank No. HM046516.1) was PCR-amplified from cDNA of PK-15 cells using a specific primer pair and cloned into the pcDNA3.1-DsRed vector fused to the C-terminal of DsRed coding sequence. All primers were presented in Table S1. All small interfering RNAs (siRNAs) against CaMKKβ, AMPK, CaMKI, and WIPI1 were purchased from Genepharma (Shanghai, China) and shown in Table S2. Scrambled, negative control siRNA (siNC) was also included. Delivery of plasmids and siRNAs into PK-15 cells was performed by using lipofectamine 2000 (Invitrogen) according to the manufacturer’s instructions.

### 2.3. Virus Infection, Treatments with Chemicals and Transfection

The PK-15 or PK-15/EGFP-LC3 cells were grown to 60% confluence in complete MEM at 37 °C and 5% CO_2_. Cells were then infected with PCV2 at a multiplicity of infection (MOI) of one and incubated at 37 °C and 5% CO_2_ for the indicated time points after treatment with different chemicals at 6 hours post-infection (hpi). All chemicals were added at the indicated concentrations as specified in corresponding figures with mock-infected cells or cells treated with the same level of the solvent dimethyl sulfoxide (DMSO) (Sigma, St. Louis, MO, USA) as controls. The chemicals used included: mTOR inhibitor rapamycin (Rapa) (Merck, Darmstadt, Germany), CaMKKβ inhibitor 7-Oxo-7H-benzimidazo(2,1-a)benz(de)isoquinoline-3-carboxylic acid acetate acid (STO-609) (Sigma), CaMKI inhibitor 2-(*N*-(2-hydroxyethyl))-*N*-(4-methoxybenzenesulfonyl)amino-*N*-(4-chlorocinnamyl)-*N*-methylamine (KN93) (Selleck Chemicals LLC, Houston, TX, USA), Sarcoplasmic/endoplasmic reticulum Ca^2+^- adenosine triphosphatase (SERCA) inhibitor thapsigargin (TG) (Abcam, Cambridge, UK), phosphoinositide 3-kinase (PI3K) inhibitor wortmannin (WM) (Sigma), IP3R inhibitor 2-aminoethoxydiphenyl borate (2-APB) (Sigma), and IP3R activator d-myo-inositol 1,4,5-tris-phosphate trisodium salt (Sigma).

To investigate the effects of PCV2 infection on the levels of cytosolic Ca^2+^, intracellular inositol 1,4,5-trisphosphate (IP3) and phosphorylated phospholipase C-gamma (p-PLC-γ), PK-15 cells were infected with PCV2 (MOI ≈ 1) and collected at 12, 24 and 36 hpi for cytosolic Ca^2+^ measurement by flow cytometry, p-PLC-γ by Western blotting, and intracellular IP3 by enzyme-linked immunosorbent assay (ELISA). Alternatively, cells were infected with PCV2 for 6 h, transfected with pcDNA3-TN-XXL and incubated for additional 30 h before being subjected to FRET detection. IP3 (10 mM) was used as positive control. The PCV2-infected cells were also incubated in the presence of IP3 (10 mM) or 2-APB (100 μM) to examine the effects of these two chemicals on PCV2-induced cytosolic Ca^2+^ levels by flow cytometry.

For gene silencing experiments, cells were infected with PCV2 (MOI ≈ 1) at 12 h post-transfection (hpt) of siRNAs (siCaMKKβ, siWIPI1, siCaMKI or siAMPK). siNC were used as controls. Cells were treated, where appropriate, for indirect immunofluorescence assay (IFA), Western blotting, virus titration, or viral DNA quantification using the methods below.

### 2.4. Western Blotting

Western blotting was performed to detect changes of the expression of target molecules in PCV2-infected cells treated with chemicals or siRNAs, or in PK-15 cells expressing Cap, or its truncated versions. Briefly, cells at the indicated time points were harvested by using a pre-cooled lysis buffer (Beyotime, Hangzhou, China) supplemented with protease inhibitor cocktail (Roche Diagnostics GmbH, Mannheim, Germany). The cell lysates were centrifuged and the supernatant samples were collected. Protein concentration in the samples were quantified using a bicinchoninic acid assay kit (MultiSciences, Hangzhou, China). The proteins were separated in 12% sodium dodecyl sulfate- polyacrylamide gel electrophoresis (SDS-PAGE) after 10-min boiling in the presence of loading buffer and electro-transferred onto polyvinylidene difluoride (PVDF) membranes (Millipore, Billerica, MA, USA). The blots were blocked for 1 h at room temperature in Tris-buffered saline (25 mM Tris at pH 7.5, 150 mM NaCl) containing 0.05% Tween 20 and 5% skim milk, and then incubated overnight at 4 °C with the following primary antibodies: mouse monoclonal anti-Cap was produced in our laboratory; mouse monoclonal anti-β-actin (MultiSciences, Hangzhou, China); rabbit polyclonal, IgG anti-LC3B (Sigma); rabbit polyclonal anti-CaMKI (phospho-Thr177) (p-CaMKI) (Santa Cruz, Dallas, TX, USA); rabbit monoclonal anti-CaMKKβ, anti-WIPI1 and anti-CaMKI (Abcam); rabbit monoclonal anti-AMPKα (phospho-Thr172) (p-AMPK), anti-AMPKα and anti-PLC-γ (phospho-Ser1248) (p-PLC-γ) from Cell Signaling Technology (CST, Boston, MA, USA). The blots were washed five times with Tris buffered saline with Tween 20 (TBST) and incubated for 1 h at 37 °C with goat anti-rabbit or goat anti-mouse antibodies conjugated to horseradish peroxidase (HRP) (KPL, Gaithersburg, MD, USA). The blots were visualized by using SuperSignal West Pico chemiluminescent substrate (Thermo, Marina, CA, USA) and images were captured in a Gel 3100 chemiluminescent imaging system (Sagecreation, Beijing, China). The protein bands were analyzed with the Quantity One software (Bio-Rad, Hercules, CA, USA) and abundance of target proteins in various treatments was expressed relative to those under mock conditions.

### 2.5. Indirect Immunofluorescence, and Quantification of Virus Titer and Viral DNA

To determine whether CaMKKβ is involved in PCV2 replication, PK-15 cells were infected with PCV2 (MOI ≈ 1) and cultured for 72 h in the presence of CaMKKβ inhibitor STO-609 (10 mM) or siCaMKKβ. The cell samples were subjected to immunofluorescence using mouse monoclonal anti-Cap antibody on the inverted fluorescence microscope X81 (Olympus, Tokyo, Japan). Treated cells, as described above, were incubated for 36 and 72 h for quantification of virus titers and viral DNA copies by quantitative PCR (qPCR) as described previously [7,37].

### 2.6. Confocal Microscopic Analysis of WIPI1 Puncta and WIPI1-LC3 Colocalization

For quantification of WIPI1 puncta and WIPI1-LC3 colocalization, PK-15 or PK-15/EGFP-LC3 cells were cultured in Petri dishes (10 mm in diameter) and infected with PCV2 at MOI ≈ 1. After incubation for 6 h at 37 °C and 5% CO_2_, the infected cells were transfected with pcDNA3.1-DsRed-WIPI1. The culture medium was replaced at 6 hpt with fresh MEM containing STO-609 (10 μM) or KN93 (2 μM) and continued to incubate for additional 24 h (total 36 hpi). TG (0.5 μM) or WM (1 μM) were used as positive and negative control, respectively [30]. Cells were washed with phosphate buffered saline (PBS), fixed and permeabilized with 80% cold acetone at −20 °C for 20 min, and washed again with PBS. Cell nuclei were stained with 4′, 6′-diamidino-2-phenylindole dihydrochloride (DAPI) (Invitrogen). Images of treated cells were captured by confocal microscopy (FV1000, Olympus, Markham, ON, Canada). WIPI1 puncta or WIPI1/EGFP-LC3 colocalization per cells was quantified from at least 50 cells/experiment from three independent experiments [39].

### 2.7. Analysis of Cytosolic Ca^2+^ by Flow Cytometry and FRET

To explore if PCV2 infection could perturb Ca^2+^ homeostasis of cells as part of the signaling components, cytosolic Ca^2+^ level in PCV2-infected PK-15 cells was measured by flow cytometric method. Cells were washed twice with Hanks’ balanced salt solution (HBSS, Beyotime), loaded with the Ca^2+^ indicator dye Fluo 3-AM (2.5 μM) and Pluronic F-127 (0.02%) (Dojindo, Shanghai, China) and incubated for 45 min at 37 °C. Cells were then washed twice and suspended in 1 mL HBSS for flow cytometry (Becton Dickinson, San Jose, CA, USA). Fluorescence intensity was collected at 488 nm (excitation) and 530 nm (emission) under the logarithmic mode. The fluorescence intensities of various treatments were expressed relative to those under mock conditions.

For analysis of cytosolic Ca^2+^ by FRET, the PCV2-infected cells were imaged using a confocal microscope. FRET efficiency was calculated with the acceptor photobleaching method according to the manufacturer’s instructions (FV1000; Olympus; Markham, ON, Canada).

### 2.8. IP3 Measurement by ELISA

IP3 cleaved from phosphatidylinositol 4,5-bisphosphate (PIP2) by PLC-γ can function as the activator of IP3R in mammalians [40]. To explore if PCV2 infection could change the intracellular IP3 level, an ELISA kit (DingGuo, Beijing, China) was used for IP3 measurement according to manufacturer’s instructions. Briefly, PCV2- or mock-infected PK-15 cells in 24-well plates at indicated time points were washed twice with pre-cooled PBS, and lysed by two rounds of freezing-thawing in 500 μL PBS. The cell lysates were centrifuged and the supernatants were collected for ELISA. Data were reported as microgram of IP3 per liter (μg/L).

### 2.9. Cell Viability Measurement

Cell viability was measured with the cell counting kit-8 (CCK-8) (Beyotime). Briefly, 1 × 10^3^ cells per well were seeded in 96-well plates and incubated at 37 °C and 5% CO_2_ for 12 h. The medium was replaced with fresh complete medium containing DMSO, STO-609 (10 μM), KN93 (2 μM), TG (0.5 μM), WM (1 μM), IP3 (10 mM), or 2-APB (100 μM), or transfected with siCaMKKβ, siAMPK, siCaMKI, siWIPI1, or siNC. The plates were incubated for 36 h. CCK-8 (10 μL) was added to each well and incubated for 2 h. Absorbance at 450 nm was measured on the SpectraMax M2 spectrophotometer (Molecular Devices, Sunnyvale, CA, USA). Viability (%) of treated cells was expressed relative to control cells treated with DMSO or siNC.

### 2.10. Statistical Analysis

All results were presented as means ± standard error of the mean (SEM) from at least three independent experiments and analyzed by using the Student’s *t*-test. The differences were considered significant when the *p*-value (*p*) was < 0.05 (*) or < 0.01 (**).

## 3. Results

### 3.1. CaMKKβ Is the Upstream Activator of AMPK in PCV2-Induced Autophagy

**Figure 1:**
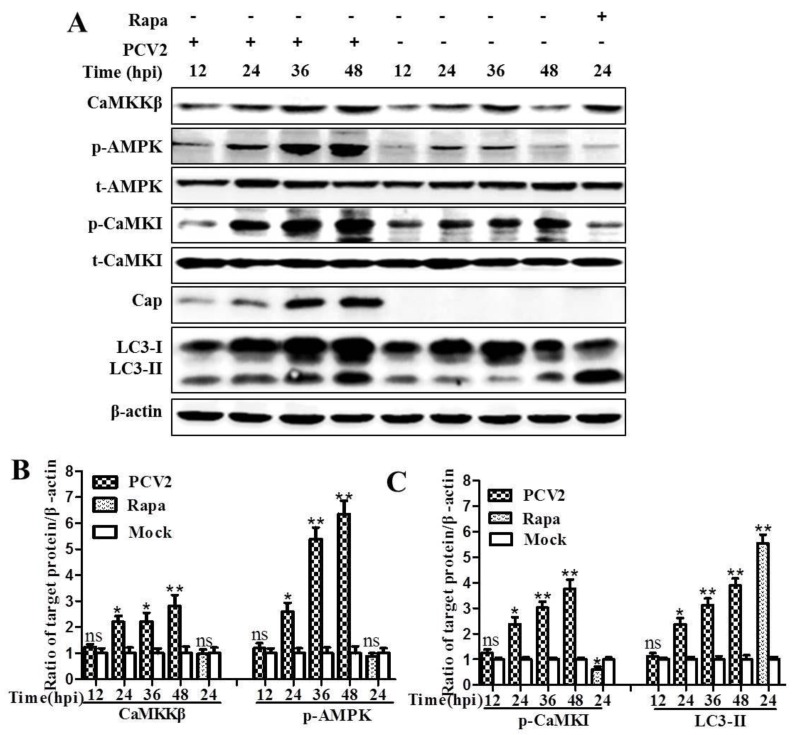
Porcine circovirus 2 (PCV2) infection activated calcium/calmodulin-dependent protein kinase kinase-beta (CaMKKβ) and its substrates calcium/calmodulin-dependent protein kinase I (CaMKI) and 5′ adenosine monophosphate-activated protein kinase (AMPK), as well as increased lipidation of microtubule-associated microtubule-associated protein 1 light chain 3 (LC3-II). PK-15 cells were infected with PCV2 (multiplicity of infection (MOI) ≈ 1) or mock-infected for the indicated time points. Whole cell lysates were subjected to Western blotting for CaMKKβ, phosphorylated AMPK (p-AMPK), total AMPK (t-AMPK), phosphorylated CaMKI (p-CaMKI), total CaMKI (t-CaMKI), viral capsid protein (Cap), and LC3-II. (**A**) Representative images of Western blotting for target proteins of cells at 12, 24, 36 and 48 hours post-infection (hpi); (**B**) ratios of CaMKKβ and p-AMPK to β-actin; (**C**) ratios of p-CaMKI and LC3-II to β-actin. Ratios of targeted proteins to β-actin were normalized to mock infection set at 1.0. Data are reported as the mean ± SEM of three independent experiments (ns, *p* > 0.05; * *p* < 0.05; and ** *p* < 0.01).

We have found that PCV2 induced autophagy via the AMPK/ERK/TSC2/mTOR pathway in PK-15 cells [37]. Here we further show that PCV2 infection significantly upregulated CaMKKβ and the phosphorylated forms of its substrate molecules AMPK (p-AMPK) and CaMKI (p-CaMKI) at different time points from 24 hpi, as compared to the mock-infected control (Figure 1A–C; *p* < 0.05 or *p* < 0.01). Significant lipidation of LC3 (LC3-II) was also observed from 24 hpi (Figure 1A,C; *p* < 0.05 or *p* < 0.01).

**Figure 2:**
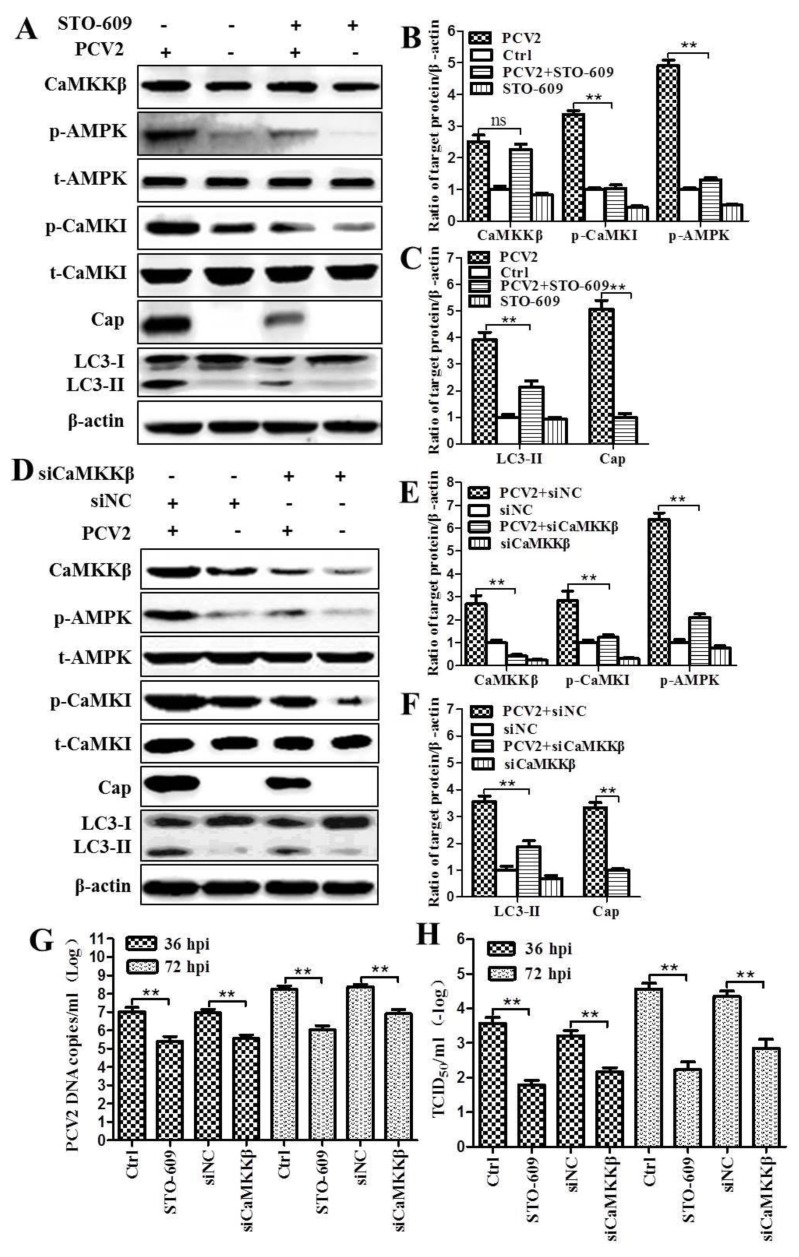
Inhibition of CaMKKβ by STO-609 or RNA silencing downregulated p-AMPK, p-CaMKI, and LC3-II as well as reduced replication of PCV2. PK-15 cells were infected with PCV2 (MOI ≈ 1) in the presence of STO-609 (10 μM) or siRNA targeting CaMKKβ (siCaMKKβ) for the indicated time points. Whole cell lysates at 36 hpi were subjected to Western blotting (**A**–**F**). (**A**,**D**) Representative images of Western blotting for target proteins of cells treated with STO-609 (**A**) or siCaMKKβ (**D**) at 36 hpi. (**B**,**E**) Ratios of CaMKKβ, p-AMPK and p-CaMKI to β-actin of panels A and D. (**C**,**F**) Ratios of LC3-II and viral capsid (Cap) protein to β-actin as represented by panels A and D. PCV2-infected cells treated with STO-609 or siCaMKKβ were used for quantitative real-time PCR (**G**) and virus titer (50% tissue culture infective dose (TCID_50_)) (**H**) at 36 and 72 hpi. Ratios of targeted proteins to β-actin were normalized to mock infection set at 1.0. All statistical data are reported as the mean ± SEM of three independent experiments (ns, *p* > 0.05; * *p* < 0.05; and ** *p* < 0.01).

Next, we investigated whether PCV2-induced phosphorylation of AMPK or CaMKI could be inhibited by CaMKKβ inhibitor STO-609 or RNA silencing. Figure 2 shows that ratios of p-AMPK, p-CaMKI and LC3-II to β-actin were significantly decreased in PCV2-infected cells in the presence of STO-609 as compared to control cells (*p* < 0.01) (Figure 2A–C). There were no significant changes of total AMPK (t-AMPK) and total CaMKKβ (t-CaMKKβ) (statistical data not shown, *p* > 0.05). The levels of p-AMPK, p-CaMKI and LC3-II were also markedly reduced when CaMKKβ was knocked-down by RNA interference (*p* < 0.01) (Figure 2D–F). These results clearly indicate that the autophagic response in PCV2-infected cells is linked with upregulation of CaMKKβ and that CaMKKβ is the upstream activator of AMPK in PCV2-induced autophagy.

Since we found reduced expression of the viral Cap protein when CaMKKβ was inhibited by STO-609 or siCaMKKβ (Figure 2C,F), we further examined whether inhibition of CaMKKβ affected viral replication. Quantitative PCR and virus titration showed that treatment of PCV2-infected cells with STO-609 or siCaMKKβ resulted in significant lower viral DNA copy numbers and virus titers, presented as 50% tissue culture infective dose (TCID_50_), than the control cells at 36 and 72 hpi (*p* < 0.01) (Figure 2G,H). These observations further support our previous findings that autophagic response in PCV2-infected cells promote viral replication.

### 3.2. PCV2 Employs CaMKI/WIPI1 for Autophagy Induction Independent of AMPK

**Figure 3:**
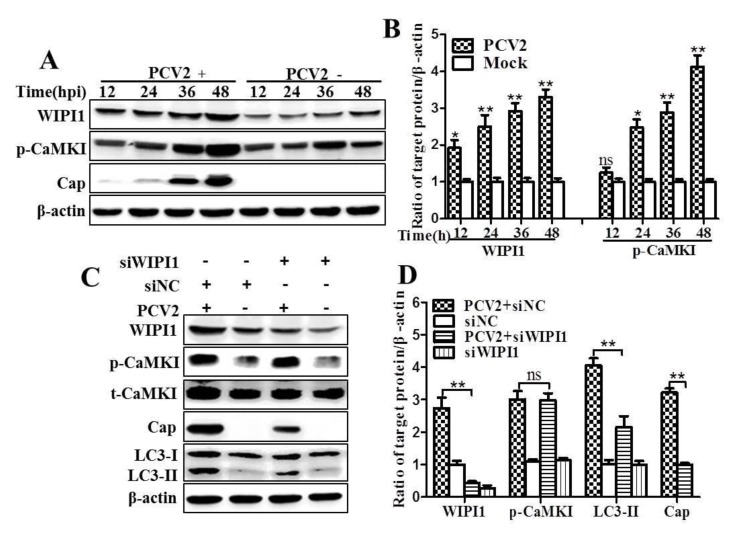
PCV2 infection upregulated Trp-Asp (WD) repeat domain phosphoinositide-interacting protein 1 (WIPI1). Knock-down of *WIPI1* downregulated LC3-II and Cap protein without affecting CaMKI. PK-15 cells were infected with PCV2 (MOI ≈ 1) in the presence of siRNA targeting WIPI1 (siWIPI1) for the indicated hpi and the whole cell lysates were subjected to Western blotting. (**A**) Representative images of Western blotting for target proteins of cells; (**B**) ratios of WIPI1, p-CaMKI to β-actin; (**C**) representative images of Western blotting for the target proteins of PCV2-infected cells treated with siWIPI1 at 36 hpi; and (**D**) ratios of WIPI1, p-CaMKI, Cap, and LC3-II to β-actin. Ratios of targeted proteins to β-actin were normalized to mock infection set at 1.0. Scrambled, negative control RNA (siNC) was included. Data are reported as the mean ± SEM of three independent experiments (ns *p* > 0.05; * *p* < 0.05; and ** *p* < 0.01).

WIPI1 acts as an essential PI3P effector at the nascent autophagosome [27], and involved in the initiation of autophagosome formation [26]. We examined if PCV2 infection activates WIPI1 via CaMKI. Figure 3 shows that WIPI1 was significantly upregulated as early as 12 hpi (*p* < 0.05) and remained higher upon further PCV2 infection than the mock controls (*p* < 0.01). p-CaMKI was upregulated significantly only from 24 hpi (*p* < 0.01) (Figure 3A,B). Next, we decided to test if WIPI1 was essential for PCV2-induced autophagy by silencing *WIPI1* gene expression. The ratios of LC3-II and Cap to β-actin were significantly decreased in PCV2-infected cells transfected with siWIPI1 as compared with scrambled siRNA (*p* < 0.01) (Figure 3C,D). However, we did not find apparent changes of p-CaMKI due to silencing of *WIPI1* (*p* > 0.05) (Figure 3C,D).

**Figure 4:**
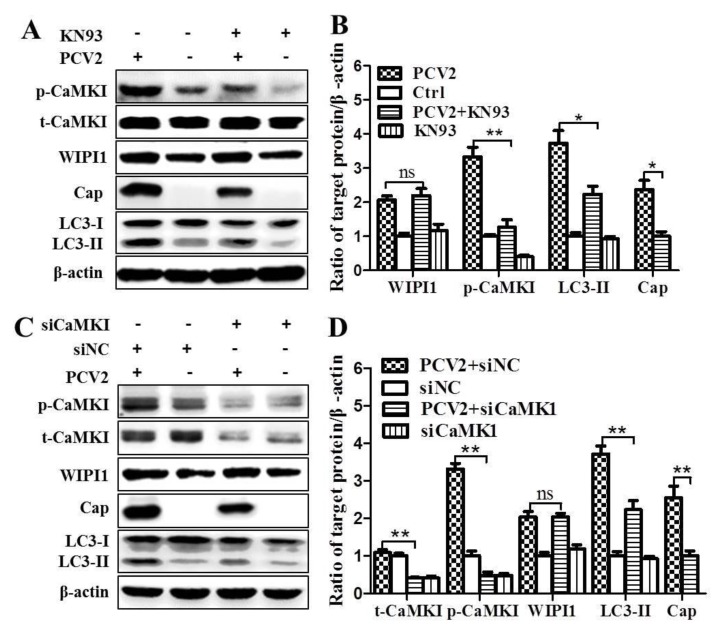
Inhibition of CaMKI by KN93 or RNA interference reduced expression of LC3-II and Cap proteins without affecting WIPI1. PK-15 cells were infected with PCV2 (MOI ≈ 1) in the presence of KN93 (2 μM) or siRNA targeting CaMKI (siCaMKI). Cells were collected at 36 hpi and the lysates were subjected to Western blotting. (**A**) Representative images of Western blotting for target proteins from cells treated with KN93; (**B**) Ratios of WIPI1, p-CaMKI, Cap and LC3-II to β-actin; (**C**) Representative images of Western blotting for target proteins from cells treated with siCaMKI; (**D**) Ratios of WIPI1, t-CaMKI, p-CaMKI, Cap and LC3-II to β-actin. Ratios of targeted proteins to β-actin were normalized to mock infection set at 1.0. Data are reported as the mean ± SEM of three independent experiments (ns *p* > 0.05; * *p* < 0.05; and ** *p* < 0.01).

To further verify if CaMKI was required for upregulation of WIPI1 during PCV2 infection, we used KN93 or siCaMKI to inhibit CaMKI. As shown in Figure 4A,B, inhibition of CaMKI with KN93 suppressed PCV2-induced conversion of LC3-I to LC3-II and Cap expression with statistical significance (*p* < 0.05) as compared with untreated control cells. However, such treatment did not affect WIPI1 protein level. Similarly, knock-down of *CaMKI* by siRNA reduced LC3 conversion and Cap expression, but had no effect on WIPI1 (Figure 4C,D, *p* < 0.01). Therefore, both WIPI1 and CaMKI might be involved in PCV2-induced autophagy.

**Figure 5:**
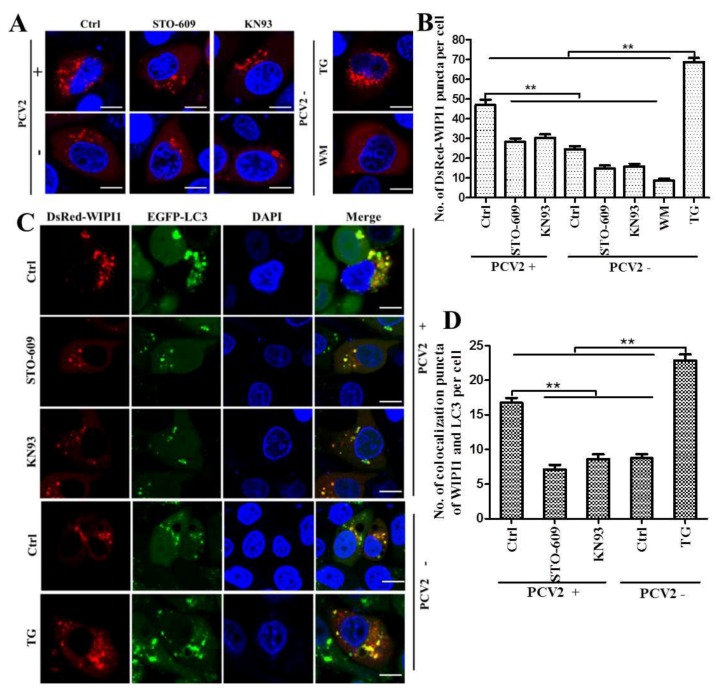
Formation of WIPI1 puncta and colocalization of WIPI1 and LC3 were increased by PCV2 infection, but decreased by treatment with STO-609 or KN93. (**A**) PCV2-infected cells were transfected with pcDNA3.1-DsRed-WIPI1 at 6 hpi, treated with STO-609 (10 μM) or KN93 (2 μM) at 6 hours post-transfection (hpt), and incubated for additional 24 h. 0.5 μM thapsigargin (TG) or 1 μM wortmannin (WM) was used as positive or negative control, respectively. Cells were examined for formation of DsRed-WIPI1 puncta under the confocal microscope (scale bar, 10 μM) at 36 hpi; (**B**) average number of DsRed-WIPI1 puncta per cell from at least 50 cells/experiment in three independent experiments; (**C**) pcDNA3.1-DsRed-WIPI1 was transiently transfected into PCV2 pre-infected PK-15 cells stably expressing EGFP-LC3 (PK15/EGFP-LC3) at 6 hpi (MOI ≈ 1) and treated with STO-609 or KN93 as above. Representative confocal images of colocalization of DsRed-WIPI1 and EGFP-LC3 in PK15/EGFP-LC3 cells are shown (scale bar, 10 μM); and (**D**) average number of colocalization of DsRed-WIPI1 and EGFP-LC3 puncta per cell from at least 50 cells/experiment in three independent experiments. Data are reported as the mean ± SEM (ns, *p* > 0.05; * *p* < 0.05; and ** *p* < 0.01).

In addition to LC3-II, WIPI1 proteins are considered to bridge PI3P production and LC3 lipidation [27]. Analysis of WIPI1 protein accumulation has been proposed as a new method to monitor autophagy in mammalian cells [30]. By confocal imaging of PCV2-infected cells expressing DsRed-WIPI1, we found that PCV2 infection triggered a significant increase in WIPI1-puncta as compared with the mock control, which could be repressed by the CaMKKβ inhibitor STO-609 or the CaMKI inhibitor KN93 (Figure 5A,B; *p* < 0.01). To further detect if WIPI1 accumulation contributed to autophagosome formation, PK-15/EGPF-LC3 cells were infected with PCV2 and then transfected with pcDNA3.1-DsRed-WIPI1. Overall, DsRed-WIPI1 and EGFP-LC3 shared similar distribution in the cytoplasm (Figure 5C). There were more colocalizations of WIPI1 and LC3 puncta in PCV2-infected cells that could be repressed by STO-609 and KN93 (Figure 5D; *p* < 0.01). These results suggest that CaMKI is required for PCV2-mediated WIPI1 accumulation, although it is not directly involved in upregulation of WIPI1 as shown above.

**Figure 6:**
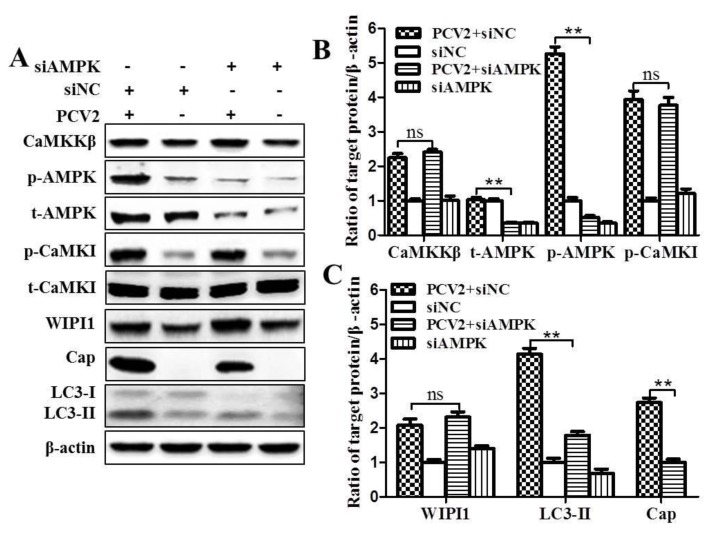
Silencing of AMPK downregulated LC3-II and Cap protein expression, but had no effect on CaMKKβ, CaMKI, and WIPI1. PK-15 cells were first transfected with siAMPK, then infected with PCV2 (MOI ≈ 1) and incubated for 36 h. The cell lysates were subjected to Western blotting. (**A**) Representative images of Western blotting for target proteins; (**B**,**C**) ratios of CaMKKβ, t-AMPK, p-AMPK, and p-CaMKI, WIPI1, LC3-II, and Cap to β-actin. Ratios of targeted proteins to β-actin were normalized to mock infection set at 1.0. Data are reported as the mean ± SEM of three independent experiments (ns, *p* > 0.05; * *p* < 0.05; and ** *p* < 0.01).

To explore if AMPK affects the expression of CaMKI and WIPI1, we knocked-down AMPK by siRNA. Figure 6 reveals that silencing was effective as shown by significant reduction of total AMPK and p-AMPK (*p* < 0.01, in comparison with siNC control). However, there were no significant changes in the levels of p-CaMKI, total CaMKI (t-CaMKI) and WIPI1 (Figure 6A–C; *p* > 0.05), indicating that AMPK had no effects on these molecules in PCV2-infected cells. LC3-II and Cap expression were significantly suppressed (Figure 6A,C; *p* < 0.01). These results demonstrate that CaMKI/WIPI1 is an additional pathway in PCV2-induced autophagy independent of AMPK. Additionally, we found that siNC did not affect expression of Cap protein and of molecules on the autophagy pathway and TG treatment did induce autophagy in PK-15 cells (Figure S1). We did not find significant changes of the viability of cells treated with STO-609, KN93, TG, WM, IP3, 2-APB, or transfection with siCaMKKβ, siAMPK, siCaMKI, siWIPI1 (Figure S2).

### 3.3. PCV2 Infection Increases Cytosolic Ca^2+^ Release from the ER via IP3R to Activate CaMKKβ

**Figure 7:**
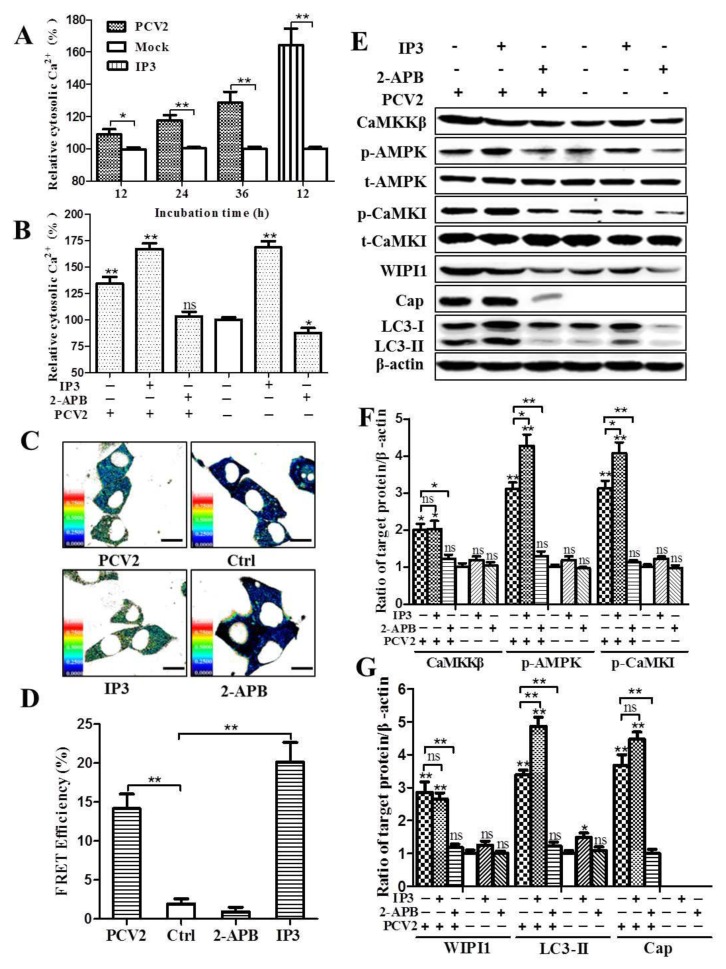
PCV2 increased cytosolic Ca^2+^ likely from the endoplasmic reticulum (ER) via inositol 1,4,5-trisphosphate receptor (IP3R) to activate CaMKKβ and its substrates. (**A**,**B**) PK-15 cells were infected with PCV2 (MOI ≈ 1) for indicated time points or treated with 10 mM inositol 1,4,5-trisphosphate (IP3) or 100 μM 2-APB at 12 hpi, and then incubated for additional 24 h before being subjected to cytosolic Ca^2+^ measurement based on chemical Ca^2+^ indicator Fluo 3-AM by flow cytometry. (**A**) Cytosolic Ca^2+^ levels relative to mock at 12, 24 and 36 hpi. (**B**) Cytosolic Ca^2+^ levels of PCV2-infected cells treated with IP3 or 2-APB at 36 hpi relative to mock-infected cells without IP3 and 2-APB treatments. (**C**,**D**) Genetically encoded Ca^2+^ indicator TN-XXL expressing plasmid (pcDNA3-TN-XXL) was transiently transfected into PK-15 cells pre-infected with PCV2 at 6 hpi (MOI ≈ 1). Mock-infected cells (Ctrl) or cells treated with IP3 (10 mM) or 2-APB (100 μM) were included for comparative purposes). (**C**) Cells were subjected to cytosolic Ca^2+^ measurement based on fluorescence resonance energy transfer (FRET) under the confocal microscope (scale bar, 10 μm) at 36 hpi. (**D**) Average FRET efficiency from at least 50 cells/experiment in three independent experiments as represented in the panel D. (**E**) The whole cell lysates with different treatments collected at 36 hpi were also subjected to Western blotting for CaMKKβ, p-AMPK and p-CaMKI, t-AMPK, t-CaMKI, WIPI1, Cap and LC3-II. (**F**,**G**) Ratios of target molecules to β-actin were normalized to mock infection without IP3 and 2-APB treatments, and set at 1.0. Data are reported as the mean ± SEM of three independent experiments (ns, *p* > 0.05; * *p* < 0.05; and ** *p* < 0.01).

Since PCV2 infection activates CaMKKβ, we assumed that Ca^2+^ signaling is probably involved. With Fluo 3-AM-based flow cytometry, we found that cytosolic Ca^2+^ in PCV2-infected cells was 6.5%, 17.6%, and 28.7% higher than the mock controls at 12, 24, and 36 hpi, respectively (Figure 7A). Treatment of PCV2-infected cells with IP3R blocker 2-APB significantly reduced cytosolic Ca^2+^ as compared to untreated PCV2-infected cells (Figure 7B; *p* < 0.01), in addition, similar effects were observed at 12 and 24 hpi (Figure S3). FRET analysis based on genetically-encoded Ca^2+^ indicator TN-XXL indicates that FRET efficiency was higher in PCV2-infected cells at 36 hpi than the mock controls (Figure 7C,D). These results suggest that PCV2 infection induces Ca^2+^ release from the ER via IP3R.

Further investigation reveals that PCV2-induced upregulation of CaMKKβ, p-AMPK, and p-CaMKI was significantly antagonized by the IP3R blocker 2-APB (Figure 7E,F; *p* < 0.05 or *p* < 0.01). Cap protein expression and LC3 lipidation were also markedly reduced (Figure 7G; *p* < 0.01). More importantly, PCV2-induced upregulation of WIPI1 could be blocked by 2-APB, indicating that increased expression of WIPI1 resulted from elevation of cytosolic Ca^2+^ during PCV2 infection. However, the IP3R activator IP3 did not have apparent effects on these molecules both in PCV2-infected and uninfected cells (Figure 7E–G).

**Figure 8:**
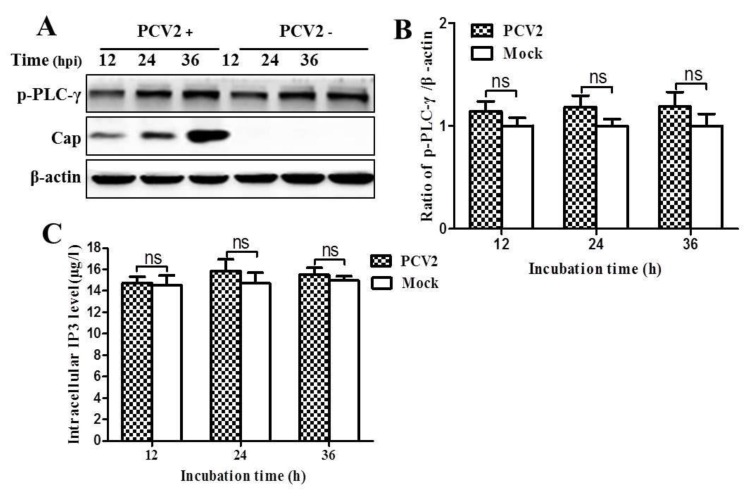
PCV2 infection did not affect phosphorylation of phospholipase C-gamma (PLC-γ), neither the level of IP3. PK-15 cells were infected with PCV2 (MOI ≈ 1) for the indicated time points and then subjected to ELISA for intracellular IP3 measurement (**A**) or to Western blotting for phosphorylated phospholipase C-γ (p-PLC-γ), Cap and β-actin (**B**). (**C**) Ratios of p-PLC-γ to β-actin as represented by the panel B. Ratios of p-PLC-γ to β-actin were normalized to mock infection set at 1.0. Data are reported as the mean ± SEM of three independent experiments (ns, *p* > 0.05; * *p* < 0.05; and ** *p* < 0.01).

We also found that PCV2 infection did not affect the levels of p-PLC-γ and intracellular IP3 at all time points measured (*p* > 0.05) (Figure 8). Taken together these results, PCV2 infection likely activates CaMKKβ and its downstream molecules by inducing Ca^2+^ release from the ER via IP3R. Such activation did not seem to involve IP3 release from PIP2 by PLC-γ.

## 4. Discussion

A number of viruses have been reported to induce autophagy via multiple pathways [17,41]. Previously, we found that PCV2 induced autophagy through activation of the AMPK/ERK/TSC2/mTOR pathway in PK-15 cells, while the mechanisms of AMPK activation are still unknown [37]. AMPK could be manipulated by a variety of viruses [42]. It can be activated by at least three factors including the increased ratio of cellular AMP/ATP or ADP/ATP, liver kinase B1 (LKB1) and CaMKKβ [43]. LKB1 activates AMPK in response to AMP/ATP or ADP/ATP elevation, while CaMKKβ phosphorylates AMPK in response to Ca^2+^ flux [44]. Here we further prove that PCV2 induces autophagy via activation of AMPK by CaMKKβ whose expression is enhanced by increased cytosolic Ca^2+^ from the ER in PCV2-infected cells.

### 4.1. AMPK Activation Is Due to Activation of CaMKKβ as a Result of Increased Cytosolic Ca^2+^ in PCV2-Infected Cells

Initially we found that PCV2 infection increased expression of CaMKKβ and phosphorylation of its downstream effectors AMPK and CaMKI as well as elevated lipidation of LC3. By chemical inhibition of CaMKKβ or by siRNA, decreased expression of CaMKKβ was apparent and accompanied with reduced levels of p-AMPK and p-CaMKI. Autophagic responses were also suppressed as shown by reduced levels of LC3-II and LC3 puncta after the treatments.

Since CaMKKβ is a versatile Ca^2+^-dependent kinase and responds to changes of intracellular Ca^2+^ [45,46], we sought to examine if PCV2 infection affected the cytosolic Ca^2+^ levels. Flow cytometry and FRET analysis showed significant elevation of cytosolic Ca^2+^ in PCV2-infected cells. This is consistent with a recent report that PCV2 infection could induce increased cytosolic Ca^2+^ in piglet lymphocytes [36]. Inhibition of IP3R by 2-APB led to apparent reduction of cytosolic Ca^2+^ together with marked decrease of CaMKKβ, p-AMPK, p-CaMKI, and LC3-II, suggesting that these molecules are linked in response to changes of Ca^2+^ levels in PCV2-infected cells. This is similar to rotavirus which was found to utilize its viroporin NSP4 protein to release Ca^2+^ from the ER into the cytoplasm and activate CaMKKβ/AMPK signaling to initiate autophagy [35]. Other viruses manipulate AMPK in different ways. Rift Valley fever virus (RVFV) employed LKB1 as the positive regulator to activate AMPK, whereas Epstein-Barr virus (EBV) inhibited the LKB1-AMPK pathway through phosphorylation of LKB1 at Ser428 with subsequent suppression of AMPK [47,48].

### 4.2. CaMKI and WIPI1 Are Activated by CaMKKβ in PCV2-Infected Cells and Involved in Autophagy

We showed that PCV2 infection could upregulate the expression of WIPI1 independent of CaMKI because siCaMK1 or chemical inhibition did not affect the WIPI1 level. Upregulation of WIPI1 might be related to increased cytosolic Ca^2+^ since inhibition of IP3R by 2-APB was accompanied with downregulation of WIPI1. However, both WIPI1 and CaMKI are required for PCV2-induced autophagy since silencing of either molecule resulted in reduced lipidation of LC3. Chemical inhibition of CaMKKβ or CaMKI diminished the level of colocalization of WIPI1-LC3. These results suggest that CaMKI does not affect WIPI1 expression but is involved in its aggregation in PCV2-induced autophagosome formation. This is in general agreement with a recent study showing that CaMKKβ/CaMKI positively contributes to autophagy onset through stimulating WIPI1 [26].

### 4.3. CaMKKβ Is Involved in Positive Regulation of PCV2 Replication

Some viruses exploit the autophagic responses to benefit their replication [17,41]. Our previous report has shown that PCV2 replication is enhanced as a result of autophagy. Here, we further show that CaMKKβ exerts a positive role in PCV2 replication since its inhibition by siCaMKKβ or STO-609 significantly reduced virus titers, viral DNA copies and Cap protein expression. However, the effects of these upstream molecules on viral replication could be indirect.

**Figure 9:**
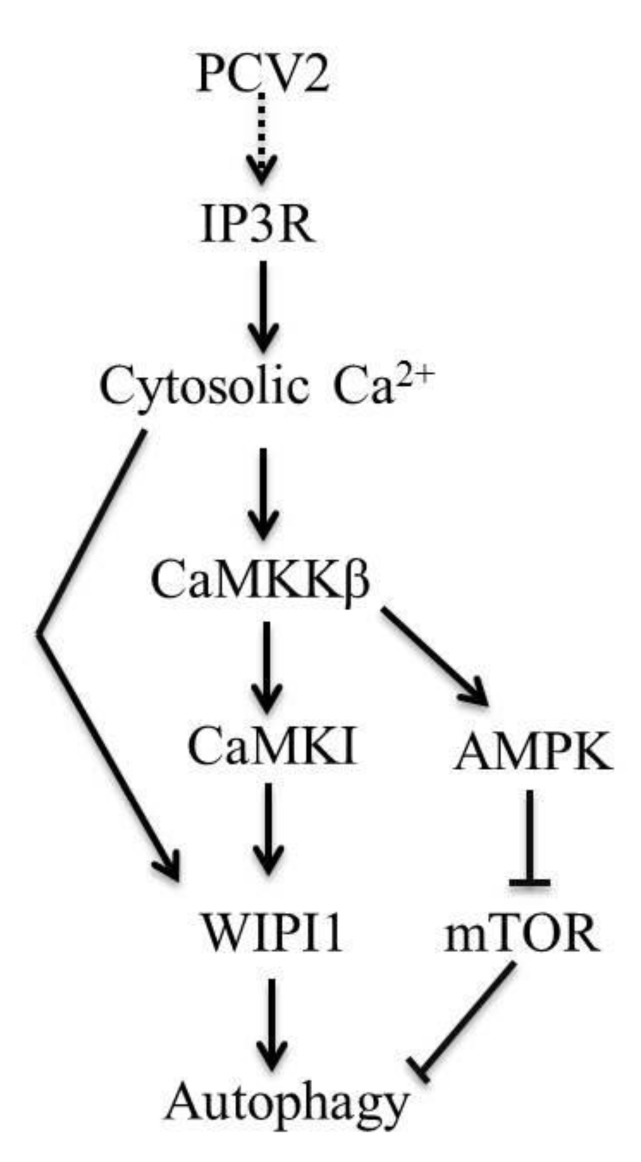
A proposed model of Ca2+ signaling in PCV2-induced autophagy in PK-15 cells. PCV2-nduced autophagy likely results from activation of IP3R and elevation of cytosolic Ca2+, which then upregulates CaMKKβ with subsequent activation of its downstream molecules AMPK and CaMKI. Two branches of the CaMKKβ signaling pathway are involved: CaMKKβ/AMPK/extracellular signal-regulated kinase (ERK)/tuberous sclerosis complex 2 (TSC2)/mammalian target of rapamycin (mTOR) and CaMKKβ/CaMKI/WIPI1 in PCV2-infected cells.

Therefore, we propose a model that PCV2 infection, activates IP3R to trigger elevation of cytosolic Ca^2+^ which then activates CaMKKβ and its downstream molecules with eventual induction of autophagy (Figure 9). Two branches of the CaMKKβ signaling pathway are involved: CaMKKβ/AMPK/ERK/TSC2/mTOR and CaMKKβ/CaMKI/WIPI1 in PCV2-infected cells. These findings, together with our previously published work [7,37], may provide a better understanding of PCV2 pathogenesis. Further study may be directed at the mechanisms of Ca^2+^ release from the ER via IP3R during PCV2 infection: is it the result of direct interaction of PCV2 with IP3R, of competition against IP3R-inhibitory proteins such as B-cell lymphoma 2 (Bcl-2) family proteins or of downregulation of IP3R-inhibitory proteins?

## 5. Conclusions

PCV2 activates CaMKKβ to initiate autophagy in PK-15 cells. The CaMKKβ/CaMKI/WIPI1 pathway is involved in autophagy induction during PCV2 infection independent of the AMPK/ERK/TSC2/mTOR pathway. Such understanding of the principles of virus-induced autophagy could provide insights into the mechanisms of PCV2 infection and antiviral strategies. Further research is required to examine if PCV2 interacts directly with IP3R or indirectly with the molecules that antagonize IP3R activity responsible for increased cytosolic Ca^2+^ both in PK-15 cells and in PCV2-targeted primary cells from pigs, such as T cells or macrophages.

## Supplementary Materials

The following are available online at www.mdpi.com/1999-4915/8/5/135/s1, Figure S1: Scrambled siRNA (siNC) did not affect expression of porcine circovirus 2 (PCV2) capside (Cap) protein and of molecules related to autophagy activation, Figure S2: Pharmacological treatments siRNA knock-down did not affect cell viability, Figure S3: PCV2 infection increased cytosolic Ca^2+^ likely from the endoplasmic reticulum (ER) via inositol 1,4,5-trisphosphate receptor (IP3R), Table S1: Primers used for cloning and quantitative real-time PCR, Table S2: Small interfering RNAs (siRNAs) used in this study.
