# Supplementary material for: Porcine Circovirus Type 2 Activates CaMMKβ to Initiate Autophagy in PK-15 Cells by Increasing Cytosolic Calcium
Source: Viruses. 2016 May 20;8(5):135. doi: 10.3390/v8050135 (PMC4885090; doi:10.3390/v8050135)
Supplement: Supplementary file 1 [file viruses-08-00135-s001.pdf]

Supplementary Materials: Porcine Circovirus Type 2 Activates CaMMKβ to Initiate Autophagy in PK-15 Cells by Increasing Cytosolic Calcium

Yuanxing Gu, Baozhu Qi, Yingshan Zhou, Xiaowu Jiang, Xian Zhang, Xiaoliang Li and Weihuan Fang

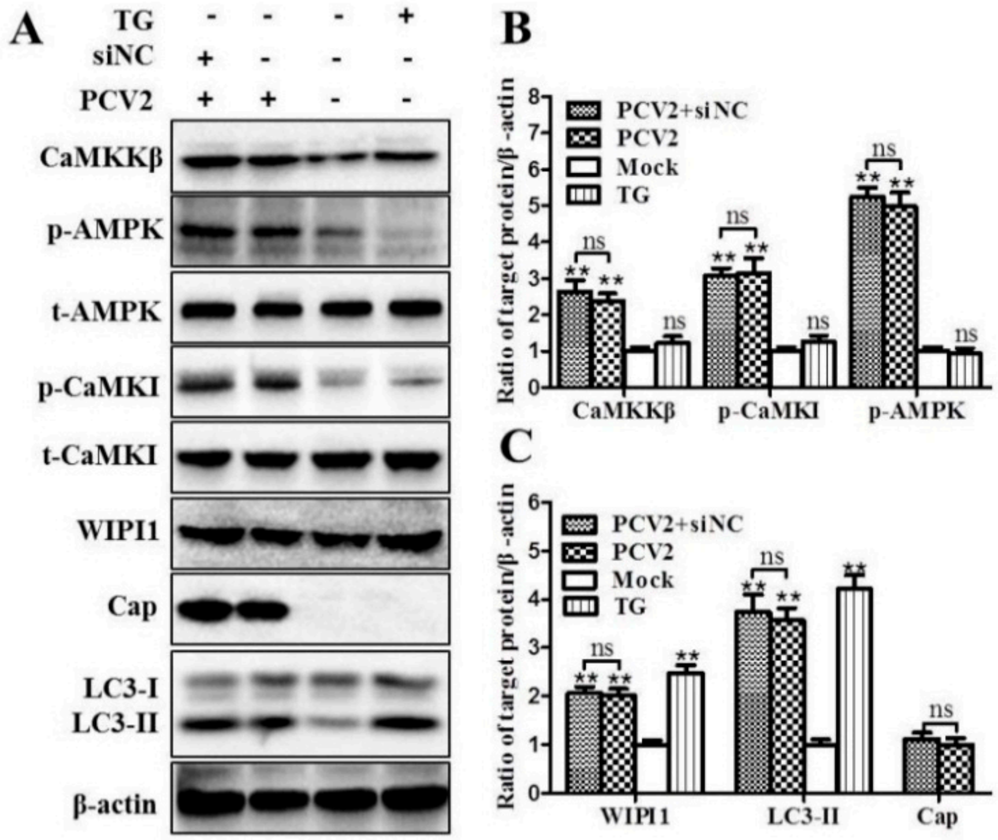

Figure S1. Scrambled siRNA (siNC) did not affect expression of porcine circovirus 2 (PCV2) capsid (Cap) protein and of molecules related to autophagy activation**.** PK-15 cells were infected with PCV2 (multiplicity of infection (MOI) ≈ 1) in the presence of siNC or 0.5 μM thapsigargin (TG). Cells were collected at 36 hours post-infection (hpi) and the lysates were subjected to Western blotting. (**A**) Representative images of Western blotting for target proteins; (**B**,**C**) ratios of calcium/calmodulin-dependent protein kinase kinase-beta (CaMKKβ), phosphorylated 5' adenosine monophosphate-activated protein kinase (p-AMPK), total AMPK (t-AMPK), phosphorylated calcium/calmodulin-dependent protein kinase I (p-CaMKI), total CaMKI (t-CaMKI), Trp-Asp (WD) repeat domain phosphoinositide-interacting protein 1 (WIPI1), Cap and microtubule-associated protein 1 light chain 3 (LC3-II) to β-actin. Ratios of targeted proteins to β-actin were normalized to mock infection set at 1.0. Data are reported as the mean ± SEM of three independent experiments (ns, $p > 0.05$; * $p < 0.05$; and ** $p < 0.01$).

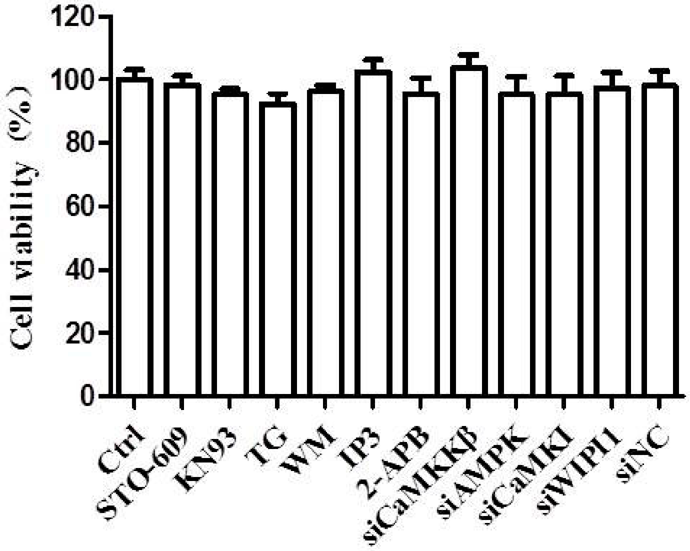

Figure S2. Pharmacological treatments siRNA knock-down did not affect cell viability. Cell viability was determined by cell counting kit-8 (CCK-8) after treatment of the PK-15 cells with STO-609 (10 μM), KN93 (2 μM), TG (0.5 μM), wortmannin (WM) (1 μM), inositol 1,4,5-trisphosphate (IP3) (10 mM), 2-APB (100 μM), or transfection with siRNA targeting calcium/calmodulin-dependent protein kinase kinase-beta (siCaMKKβ), 5' adenosine monophosphate-activated protein kinase (siAMPK), calcium/calmodulin-dependent protein kinase I (siCaMKI), Trp-Asp (WD) repeat domain phosphoinositide-interacting protein 1 (siWIPI1), or scrambled siRNA (siNC) for 36 h. Percent of cell viability is expressed as the mean ± SEM to untreated controls of three independent experiments.

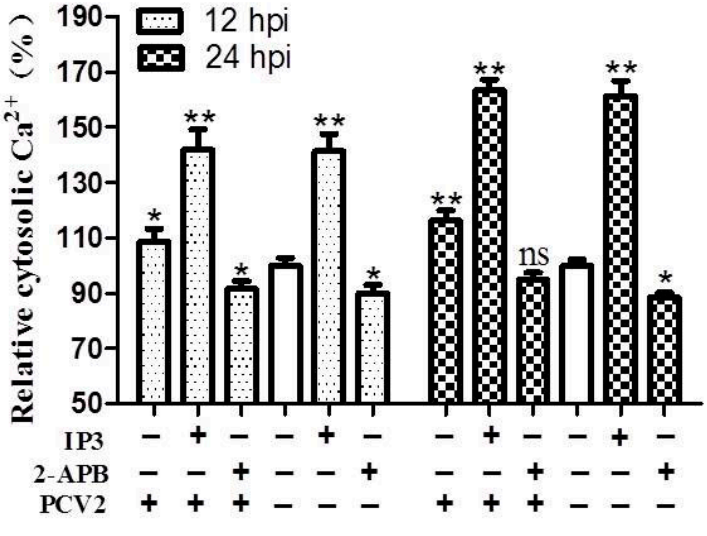

Figure S3. PCV2 infection increased cytosolic Ca^{2+} likely from the endoplasmic reticulum (ER) via inositol 1,4,5-trisphosphate receptor (IP3R). PK-15 cells were infected with PCV2 (MOI ≈ 1), treated with IP3 (10 mM) or 2-APB (100 μM) at 6 hpi, and then incubated for additional 6 h or 18 h before being subjected to cytosolic Ca^{2+} measurement by flow cytometry using the chemical Ca^{2+} indicator Fluo 3-AM. Cytosolic Ca^{2+} levels of PCV2-infected cells treated with IP3 or 2-APB at 12 or 24 hpi were shown relative to mock-infected cells without IP3 or 2-APB treatments. Data are reported as the mean ± SEM of three independent experiments (ns $p > 0.05$, * $p < 0.05$ and ** $p < 0.01$).

Table S1. Primers used for cloning and quantitative real-time PCR.

Gene Product	Sense Primer (5′ to 3′)	Antisense Primer (5′ to 3′)
DsRed-linker	TAGGATCCGCCACCATGGCCTCCT CCGAGGAC	TCCTCCGCTTCCTCCCAGGAACAG GTGGTGGCG
Linker-WIPI1	GGAGGAAGCGGAGGAATGGAGGC CGAGGCCGCGGGC	CCCTCGAGCCAAAACCACCTGACA GGGA
DsRed-WIPI1	TAGGATCCGCCACCATGGCCTCCT CCGAGGAC	CCCTCGAGCCAAAACCACCTGACA GGGA
qPCR-Cap	CGCTCTGTGCCCTTTGAATAC	GTGAGGGCTGTGGCCTTTGTTAC
qPCR-GAPDH	AAGTTCCACGGCACAGACAAGG	CACAACATACGTAGCACGAGCAT

Cap: capsid; DsRed: *Discosoma sp*. red fluorescent protein; GAPDH: glyceraldehyde 3-phosphate dehydrogenase; WIPI1**:** Trp-Asp (WD) repeat domain phosphoinositide-interacting protein 1.

Table S2. Small interfering RNAs (siRNAs) used in this study.

Targeted Genes	Sense (5′ to 3′)	Antisense (5′ to 3′)
siCaMKKβ	CAGGAAAUUGCCAUCCUCAAGdTdT	CUUGAGGAUGGCAAUUUCCUGdTdT
siAMPK	GGUUCUCAGCUGCCUUUAUdTdT	AUAAAGGCAGCUGAGAACCdTdT
siCaMKI	CCAUCAAAUGUAUCGCCAAdTdT	UUGGCGAUACAUUUGAUGGdTdT
siWIPI1	GCUUCAAGCAACACAGAAAdTdT	UUUCUGUGUUGCUUGAAGCdTdT
siNC	UUCUCCGAACGUGUCACGUdTdT	ACGUGACACGUUCGGAGAAdTdT

AMPK: 5' adenosine monophosphate-activated protein kinase; CaMKI: calcium/calmodulin-dependent protein kinase I; CaMKKβ: calcium/calmodulin-dependent protein kinase kinase-beta; NC: scrambled, negative control; WIPI1**:** Trp-Asp (WD) repeat domain phosphoinositide-interacting protein 1.

© 2016 by the authors; licensee MDPI, Basel, Switzerland. This article is an open access article distributed under the terms and conditions of the Creative Commons by Attribution (CC-BY) license (http://creativecommons.org/licenses/by/4.0/).
